# Financial Strain, Self-Rated Health, and the Mediating Role of Health Behaviors During the Great Recession

**DOI:** 10.1093/geroni/igaa057.1272

**Published:** 2020-12-16

**Authors:** Lindsay Wilkinson

**Affiliations:** Baylor University, Waco, Texas, United States

## Abstract

Previous research has revealed the physical and mental health consequences of the economic recession that began in 2007 and ended in 2009. Despite accumulating evidence of the harmful health effects of the economic downturn on older adults, relatively little research has examined the mechanisms involved in this relationship. Moreover, most research on the financial crisis has relied on objective indicators of the recession, despite evidence demonstrating the predictive utility of subjective measures such as financial strain. Drawing on a subsample of respondents age 50 or older from the Health and Retirement Study (N = 2040), this research (1) examines whether initial financial strain and that due to the recession independently contribute to worsening self-rated health over the period of the recession and (2) investigates the role of health behaviors as mechanisms in this relationship. Using longitudinal lagged dependent variable models that adjust for pre-recession self-rated health, the results reveal that both initial and increased financial strain due to the recession were associated with worsening self-rated health between 2006 and 2009. In addition, increased financial strain during the recession was found to be associated with skipped or postponed health care visits and change in prescription drug use, such as pill splitting and reduced or skipped doses, which, in turn, were associated with decreased health ratings. The results from this study suggest two important pathways in the recession-health relationship and have implications for policies aimed at supporting older adults during future financial crises.

